# Structural interplay of the redox co-chaperone CnoX to GroEL/ES chaperonin

**DOI:** 10.26508/lsa.202603764

**Published:** 2026-08-07

**Authors:** Junhak Kim, Mingyu Jung, Soung-Hun Roh

**Affiliations:** 1 School of Biological Sciences, Seoul National University , Seoul, Republic of Korea; 2 Institute of Molecular Biology and Genetics, Seoul National University , Seoul, Republic of Korea

## Abstract

Protein folding by the bacterial chaperonin GroEL/ES relies on ATP-driven conformational cycles that promote substrate encapsulation and folding. Under oxidative stress, the redox-active co-chaperone CnoX protects oxidized proteins and associates with GroEL, yet the structural basis of its interaction with the GroEL/ES remains incompletely understood. Using single-particle cryo-electron microscopy, we resolved four distinct nucleotide-bound conformational states of CnoX-associated GroEL/ES complexes. CnoX remains tethered to GroEL through its C-terminal TPR domain despite substantial rearrangements of the GroEL apical domains. We further captured a GroEL/ES-CnoX ternary assembly in which CnoX and GroES simultaneously occupy the same GroEL ring, demonstrating that their binding sites are structurally distinct and non-overlapping. Comparison of two GroES-bound states reveals how apical-domain compaction occludes the CnoX-binding surface and coincides with loss of CnoX from the cis-ring. Together, these structures define how CnoX is accommodated and excluded across distinct GroEL/ES conformations and provide a structural framework for understanding the interplay between redox co-chaperones and chaperonin assemblies.

## Abstract (precis)

Cryo-EM structures reveal how the redox co-chaperone CnoX is accommodated during the GroEL reaction cycle and how GroES binding remodels the apical domains to promote CnoX release.

## Introduction

Proteins must fold into precise three-dimensional structures to perform their cellular functions (Anfinsen, 1973). Misfolding can lead to loss of activity, aggregation, and disruption of proteostasis, contributing to stress responses and disease conditions (Mogk et al, 2018). In the crowded cellular environment, nascent or stress-denatured polypeptides rely on molecular chaperones to prevent aggregation and ensure proper folding (Fatima et al, 2021). Among these, chaperonins represent a specialized class of ATP-dependent folding machines that encapsulate substrate proteins within a central cavity, providing a protected environment distinct from the cytosol (Horovitz et al, 2022).

The bacterial chaperonin GroEL, together with its co-chaperone GroES, constitutes the prototypical Group I chaperonin system (Horwich & Fenton, 2020). GroEL is an ∼800-kD tetradecamer composed of two heptameric rings stacked back-to-back, with each subunit containing apical, intermediate, and equatorial domains responsible for substrate recognition, conformational coupling, and ATP binding, respectively (Horwich & Fenton, 2020). GroES, a heptameric ∼70-kD cofactor, binds to the apical domains of one GroEL ring to form a folding chamber that transiently encapsulates substrate proteins (Horwich & Fenton, 2020). Through ATP binding and hydrolysis, GroEL alternates between open and closed conformations, driving iterative folding cycles within this nanocage (Horwich & Fenton, 2020). Depending on the nucleotide state, GroES association occurs either asymmetrically (“bullet” complex) or symmetrically (“football” complex), transitions coordinated by inter-ring negative allostery to optimize folding efficiency (Yan et al, 2018; Wagner et al, 2024). Extensive structural and biochemical studies have elucidated the molecular principles of these ATP-driven conformational transitions (Horwich & Fenton, 2020).

**Figure 1.**
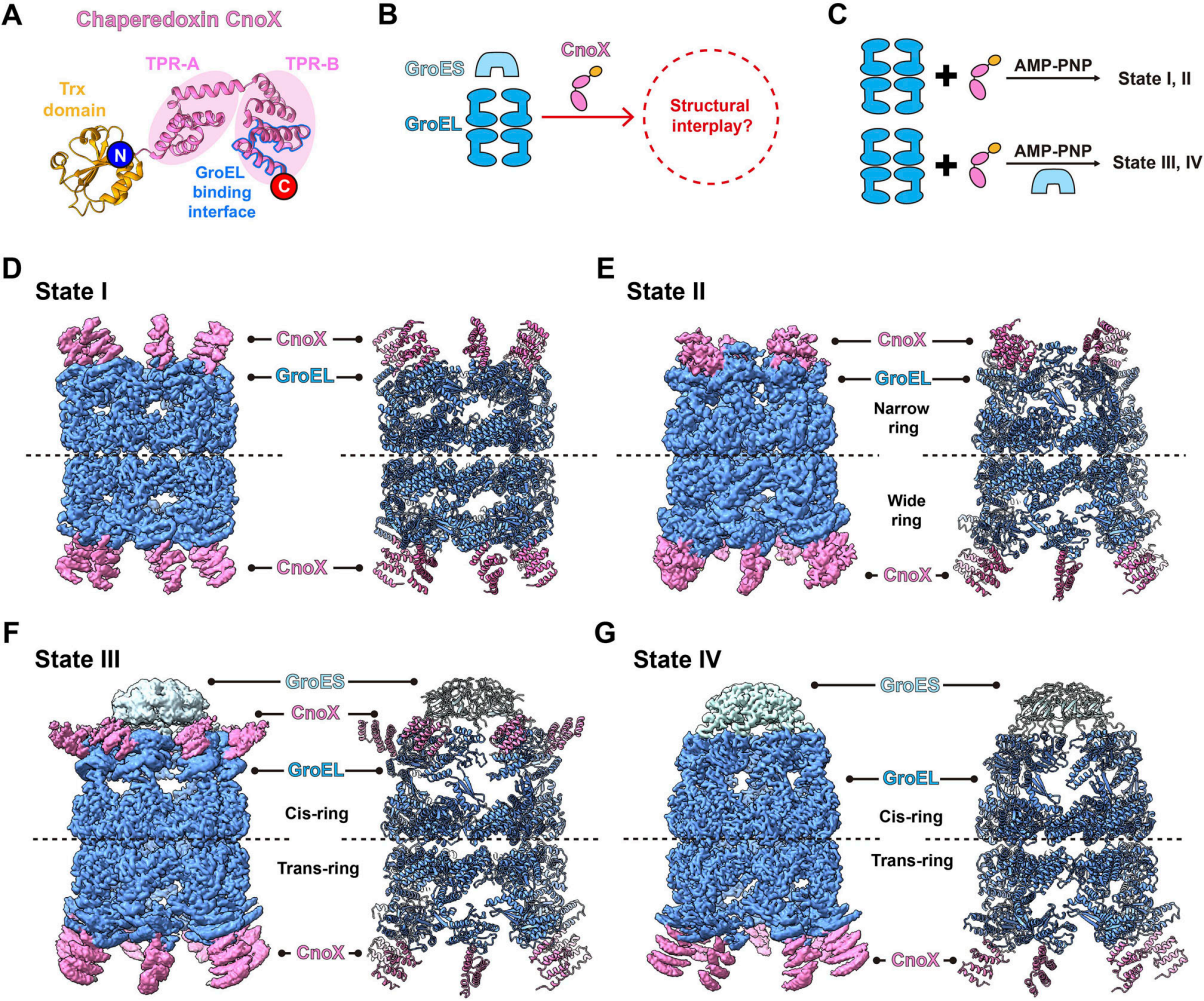
Cryo-EM structures of distinct CnoX–GroEL/ES states. **(A)** Domain architecture of chaperedoxin CnoX (PDB 3QOU). TPR domains (A, B) and the Trx domain are indicated; the GroEL-binding interface is highlighted. **(B)** Schematic illustration of CnoX association with GroEL/ES and outstanding questions regarding their structural interplay. **(C)** Experimental scheme showing reconstitution of different CnoX–GroEL/ES states in the presence of nucleotides. **(D, E, F, G)** Side views of cryo-EM density maps (left) and corresponding atomic models (right) for states I–IV. CnoX is colored pink, GroEL cornflower blue, and GroES light blue. Dashed lines indicate the equatorial plane between the rings.

However, despite its remarkable efficiency, the GroEL/ES system faces major challenges under oxidative stress, when reactive oxygen species promote cysteine oxidation and disulfide crosslinking in nascent or misfolded substrates (Ezraty et al, 2017; Santra et al, 2018). Such oxidatively damaged proteins often fail to refold productively within the canonical GroEL/ES cycle (Goemans et al, 2018a, 2018b). To counter this, bacteria use an auxiliary redox-active chaperone, CnoX, which combines a thioredoxin (Trx) domain with two tetratricopeptide repeat (TPR) domains (TPR-A and TPR-B) and directly associates with GroEL (Lin & Wilson, 2011; Goemans et al, 2018b; Dupuy et al, 2023; Fig 1A). CnoX functions as a chaperedoxin, a dual-function molecule acting both as a holdase that prevents aggregation and as a redox factor that protects and repairs oxidized cysteine residues in substrate proteins (Goemans et al, 2018b). The N-terminal Trx domain captures oxidized thiols through transient disulfide bond formation, whereas the TPR domains mediate interactions with substrates or other chaperone systems, including DnaK/DnaJ/GrpE and GroEL/ES (Kthiri et al, 2008; Goemans et al, 2018a, 2018b). Within the GroEL/ES pathway, CnoX serves as a redox quality controller that reduces oxidized substrates before their encapsulation, thereby promoting correct folding (Goemans et al, 2018b; Dupuy et al, 2023). Upon GroES binding, CnoX dissociates from GroEL, suggesting a dynamic coupling between redox repair and the ATP-driven chaperonin cycle (Dupuy et al, 2023). Despite these biochemical insights, the structural basis underlying CnoX–GroEL/ES cooperation, including how CnoX is accommodated across distinct GroEL conformations and how its association changes upon GroES binding, remains unresolved.

In this study, we used single-particle cryo-electron microscopy (cryo-EM) to define the structural basis of chaperedoxin CnoX accommodation across GroEL/ES conformations. By resolving four distinct conformational states, we show that CnoX remains associated with GroEL across structurally diverse nucleotide-bound assemblies. These structures capture the coexistence of CnoX and GroES on the same GroEL ring and reveal how GroES-associated conformational remodeling occludes the CnoX-binding surface. Together, our findings provide a structural framework for understanding how redox co-chaperones are accommodated within chaperonin assemblies.

## Results

### Cryo-EM structures of distinct CnoX–GroEL/ES assemblies

**Figure S1.**
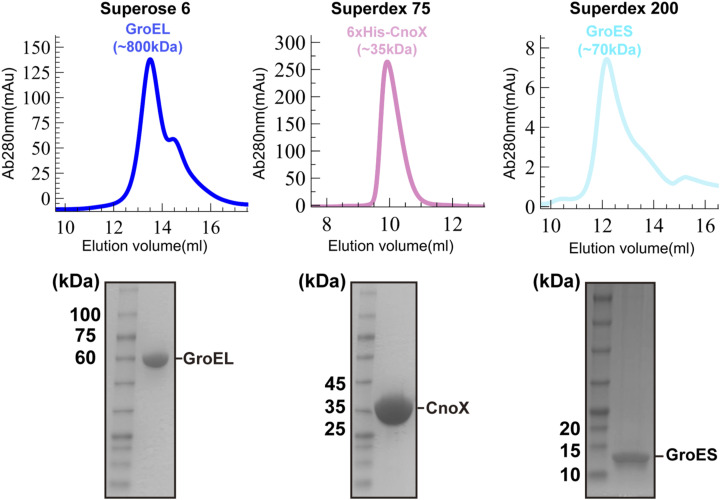
Protein purification. Size-exclusion chromatography profiles and corresponding SDS–PAGE analyses of purified GroEL (left), CnoX (middle), and GroES (right).

To investigate how CnoX is accommodated across GroEL/ES conformations (Fig 1B), we reconstituted GroEL complexes with CnoX in the presence or absence of GroES using purified proteins (Figs 1C and S1). Based on previous biochemical observations that CnoX associates with GroEL before stable GroES engagement (Dupuy et al, 2023), we used the non-hydrolyzable ATP analog adenylyl-imidodiphosphate (AMP-PNP) to stabilize GroEL/ES assemblies and enrich nucleotide-bound conformational states under substrate-free conditions (Roseman et al, 1996; Llorca et al, 1997; Sparrer et al, 1997; Dupuy et al, 2023; Liao et al, 2024) (Fig 1C). Cryo-EM analysis revealed four distinct CnoX–GroEL/ES conformational states (states I–IV) (Fig 1C–G). Across all states, additional density attributable to the TPR-B domain of CnoX decorates the apical domains of GroEL subunits, identifying the C-terminal TPR-B helix as the primary anchoring interface, whereas the Trx and TPR-A domains remain unresolved (Fig 1D–G), consistent with their reported structural flexibility (Dupuy et al, 2023).

**Figure S2.**
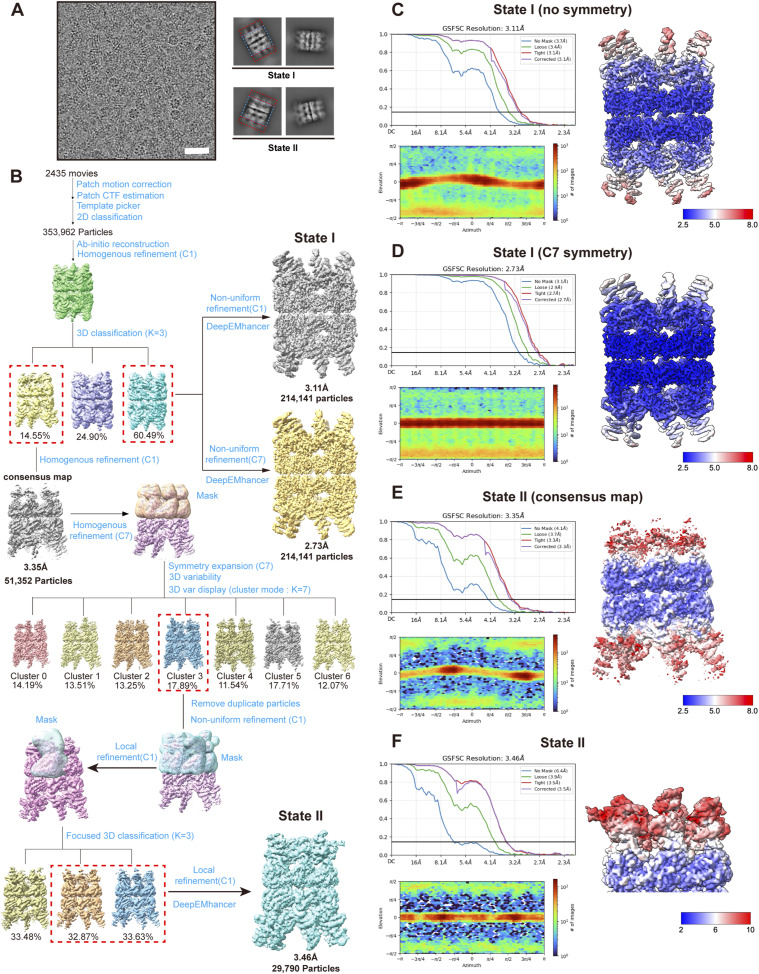
Cryo-EM image-processing workflow for state I and state II CnoX–GroEL complexes. **(A)** Representative micrograph and 2D class averages of state I and state II CnoX–GroEL complexes. Red boxes mark CnoX densities and blue boxes mark GroEL. Scale bar, 30 nm. **(B)** Overview of the single-particle processing workflow. **(C)** Local-resolution map, gold-standard FSC curve, and particle-orientation distribution of state I (C1 symmetry). **(D)** Local-resolution map, gold-standard FSC, and particle-orientation distribution of state I (C7 symmetry imposed). **(E)** Local-resolution map, gold-standard FSC, and orientation distribution of the state II consensus map. **(F)** Local-resolution map, gold-standard FSC, and orientation distribution of the state II final reconstruction.

**Figure S3.**
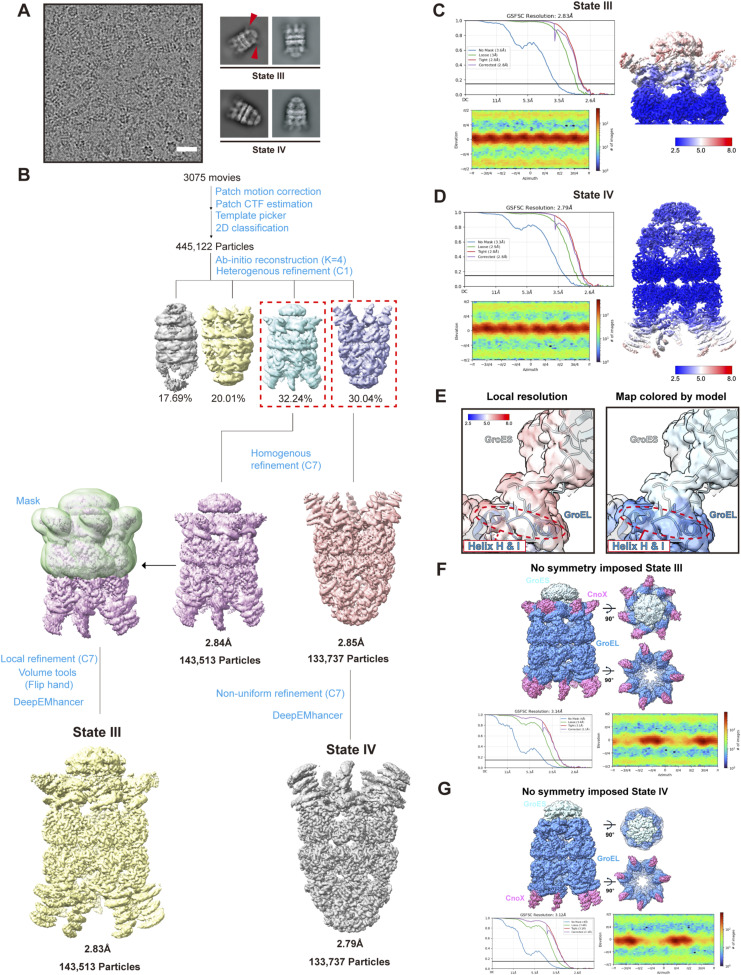
Cryo-EM image-processing workflow for state III and state IV CnoX–GroEL/ES complexes. **(A)** Representative micrograph and 2D class averages of state III and state IV CnoX–GroEL/ES complexes. The red arrow marks CnoX bound to the GroEL–GroES complex in state III. Scale bar, 30 nm. **(B)** Overview of the single-particle cryo-EM processing workflow. **(C)** Local-resolution map, gold-standard FSC curve, and particle-orientation distribution of state III (C7 symmetry imposed). **(D)** Local-resolution map, gold-standard FSC, and particle-orientation distribution of state IV (C7 symmetry imposed). **(E)** Zoomed-in view of local-resolution map, map colored by model and fitted model to the map of state III. **(F)** Cryo-EM density map, FSC curve, and particle-orientation distribution of state III (C1 symmetry). **(G)** Cryo-EM density map, FSC curve, and particle-orientation distribution of state IV (C1 symmetry).

**Figure S4.**
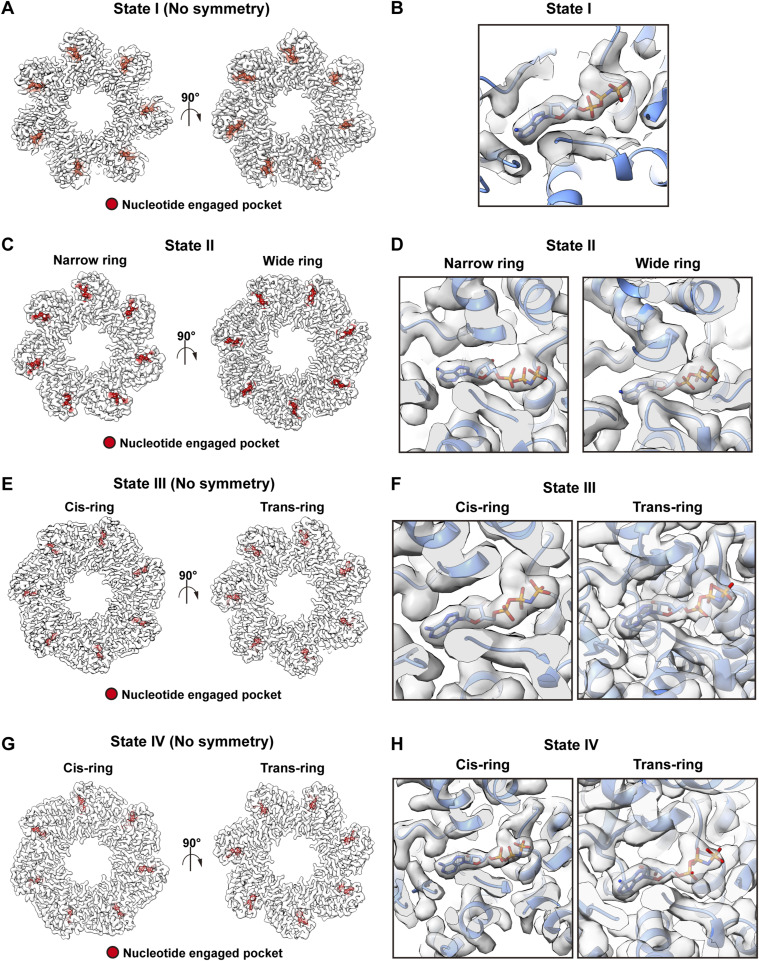
Nucleotide binding pocket analysis of distinct CnoX–GroEL/ES states. **(A)** Cryo-EM density for nucleotide-bound pockets in state I (C1 symmetry). **(B)** Close-up view of nucleotide occupancy in state I. **(C)** Nucleotide densities in the nucleotide-binding pockets of the narrow and wide rings in state II. **(D)** Close-up views of nucleotide binding in the narrow and wide rings of state II. **(E)** Cryo-EM density for nucleotide-bound pockets in the cis- and trans-rings of state III (C1 symmetry). **(F)** Close-up views of nucleotide binding in the cis- and trans-rings of state III. **(G)** Nucleotide densities in the cis- and trans-rings of state IV (C1 symmetry). **(H)** Close-up views of nucleotide binding in state IV.

States I and II correspond to CnoX–GroEL assemblies formed in the absence of GroES, reconstructed at overall resolutions of ∼3.1–3.5 Å (Figs 1D and E, S2A–F, and Table S1). State I adopts a nearly symmetric architecture with visible nucleotide densities in the binding pockets (Figs 1D and S4A and B), closely resembling a previously reported nucleotide-bound conformation (Torino et al, 2023). State II exhibits pronounced inter-ring asymmetry, with the narrow and wide rings adopting distinct conformations, structurally compatible with GroES engagement (Gardner et al, 2023; Fig 1E). States III and IV correspond to CnoX–GroEL–GroES ternary assemblies reconstructed at overall resolutions of ∼2.8 Å (Figs 1F and G, S3A–D, and Table S1). In state III, GroES engages the cis-ring, whereas CnoX remains bound to both the cis- and trans-rings, with weak and heterogeneous GroES density, consistent with a partially stabilized cis-ring conformation (Figs 1F and S3C). The GroES mobile loop was not fully resolved in state III, with local resolution of 5–8 Å in this region (Fig S3E). The interaction with GroEL helices H and I was assigned based on the density envelope and established GroEL–GroES interaction interfaces (Xu et al, 1997; Fig S3E). State IV exhibits the canonical bullet-shaped GroEL–GroES architecture, characterized by strong GroES density on the cis-ring and the absence of observable CnoX density from the same ring, whereas CnoX remains associated with all seven subunits of the trans-ring (Figs 1G and S3D). All reconstructions exhibited nucleotides clearly engaged in all nucleotide-binding pockets, confirming that they represent nucleotide-bound chaperonin assemblies (Fig S4A–H). Collectively, these cryo-EM structures reveal how CnoX is accommodated across structurally distinct GroEL/ES assemblies.

Table S1. Cryo-EM data collection, processing, and refinement statistics.

### CnoX remains accommodated across distinct GroEL apical domain conformations

**Figure 2.**
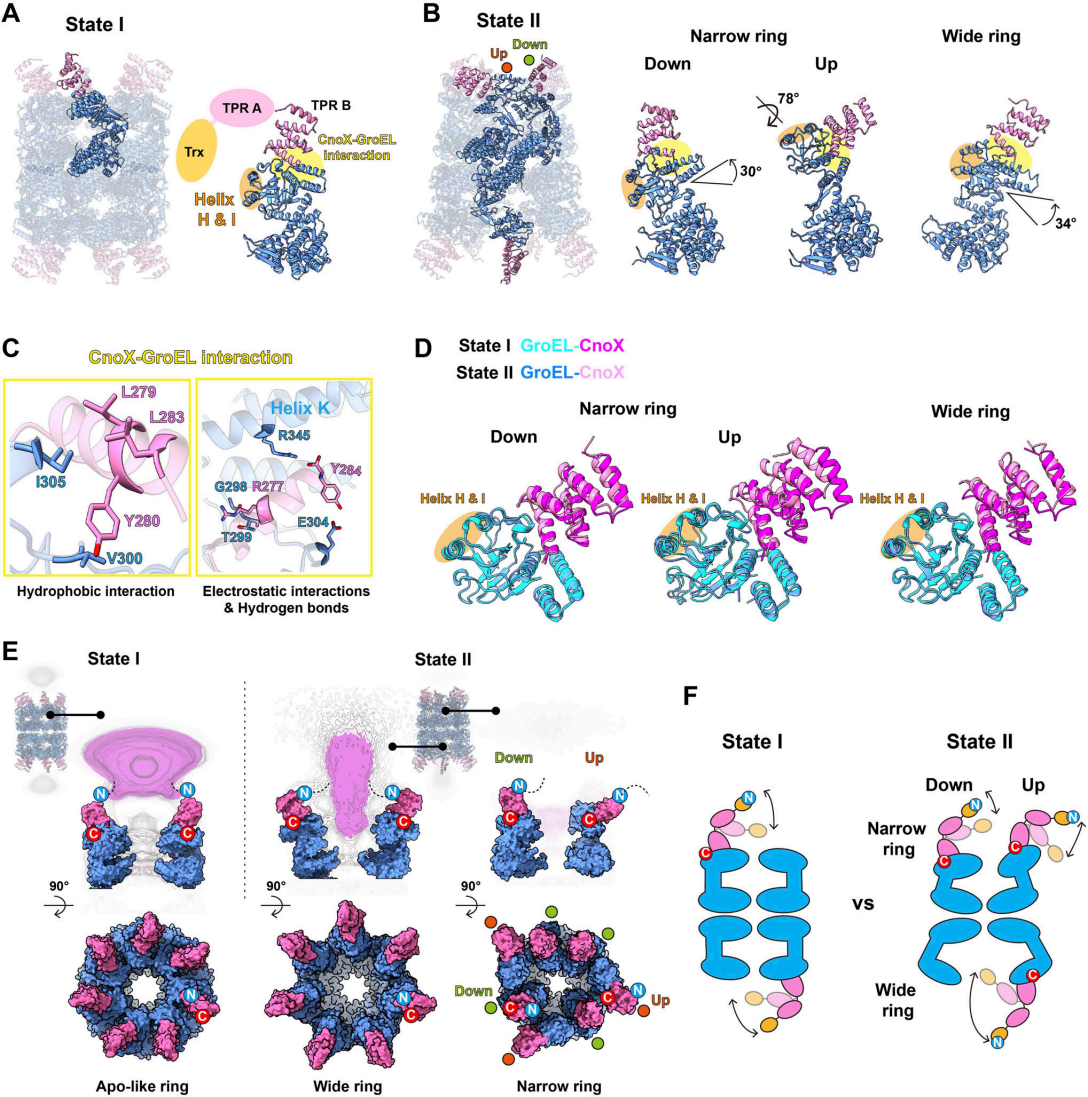
CnoX accommodation across distinct GroEL conformations. **(A)** Model of the state I CnoX–GroEL complex and a single subunit showing the CnoX-binding interface (yellow) and helices H and I (orange). **(B)** Model of the state II CnoX–GroEL complex highlighting three distinct subunit conformations (“Down,” “Up,” and wide-ring). Rotational differences relative to the state I subunit are indicated. **(C)** Close-up views of the CnoX–GroEL interface, showing hydrophobic contacts (left) and electrostatic or hydrogen-bond interactions (right). **(D)** Superposition of apical domains from states I and II. CnoX (magenta/pink) and GroEL (cyan/blue) models illustrate the conformational divergence. **(E)** Low-contour cryo-EM density maps integrated with corresponding models of states I and II. N- (blue) and C termini (red) of CnoX are marked. **(F)** Schematic representation of distinct CnoX–GroEL conformations observed in states I and II. Differences in apical-domain organization are associated with altered positioning of the unresolved N-terminal region of CnoX relative to the GroEL cavity.

**Figure S5.**
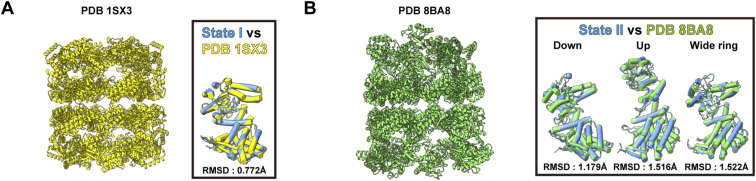
Structural comparison of state I and II with previous models. **(A)** Comparison of the state I GroEL subunit with PDB 1SX3. **(B)** Comparison of the three conformations of the state II GroEL subunits with PDB 8BA8.

State I adopted an inter-ring symmetry (Figs 1D and 2A), whereas state II exhibited pronounced asymmetry: the narrow ring displayed a mixture of “up” and “down” apical conformations among individual subunits, whereas the wide ring retained an overall symmetric organization (Figs 1E and 2B). These variations span a range of apical-domain configurations from the apo-like to more open or partially closed states (Fig 2A and B). In the narrow ring, “up” apical domains rotated upward by ∼78°, whereas “down” domains tilted upward by around 30°, repositioning helices H and I into orientations compatible with both GroES binding and substrate interaction (Fig 2B). In contrast, apical domains in the wide ring underwent a uniform ∼34° tilt, generating an open architecture unfavorable for encapsulation (Fig 2B). Throughout these large-scale conformational changes, CnoX remained anchored to the apical domains through its C-terminal TPR-B helix, maintaining a conserved hydrophobic and electrostatic interface despite GroEL ring motion (Fig 2C and D). Structural comparisons further confirmed that subunits in state I closely resembled the apo-like GroEL with nucleotides (PDB 1SX3 [Chaudhry et al, 2004]; RMSD 0.77 Å), whereas those in state II aligned with an intermediate nucleotide-bound state of GroEL that exhibits inter-ring asymmetry (PDB 8BA8 [Gardner et al, 2023]; RMSD < 2 Å) (Fig S5A–B). Together, these findings show that CnoX remains associated with GroEL across distinct apical-domain conformations without substantially altering the overall architecture of the complex.

The CnoX-binding site is spatially distinct from the canonical substrate-binding regions (helices H and I) (Fig 2A), indicating that whereas GroEL reshapes its apical domains to control substrate capture and chamber closure, CnoX remains anchored at the periphery. Although the N-terminal Trx and TPR-A domains of CnoX are highly flexible and thus unresolved at high resolution, their approximate positions could be inferred from low-contour density features (Fig 2E). Comparison of low-threshold maps across the apo-like, wide, and narrow rings revealed that the conformational differences in GroEL are associated with concerted apical-domain rearrangements that reposition these flexible CnoX domains (Fig 2E). In the apo-like ring, the apical domains adopt an upright orientation, and the associated N-terminal densities of CnoX extend upward, remaining distant from the folding chamber (Fig 2E). Upon nucleotide binding, apical domains tilt to widen the ring, creating inward-projecting low-contour densities consistent with partial insertion of the CnoX N terminus toward the chamber interior (Fig 2E). This configuration places low-contour density attributed to the flexible N-terminal region closer to the chamber entrance. Conversely, in the narrow ring—particularly in “up” subunits undergoing GroES-competent transitions—apical domains rotate upward, pulling the attached N-terminal regions outward and restricting their chamber accessibility (Fig 2E). Together, these observations suggest that CnoX remains tethered to GroEL through a conserved TPR-B interaction while its flexible N-terminal regions are repositioned in concert with apical-domain rearrangements (Fig 2F).

### GroES-associated conformational remodeling occludes the CnoX-binding surface

**Figure 3.**
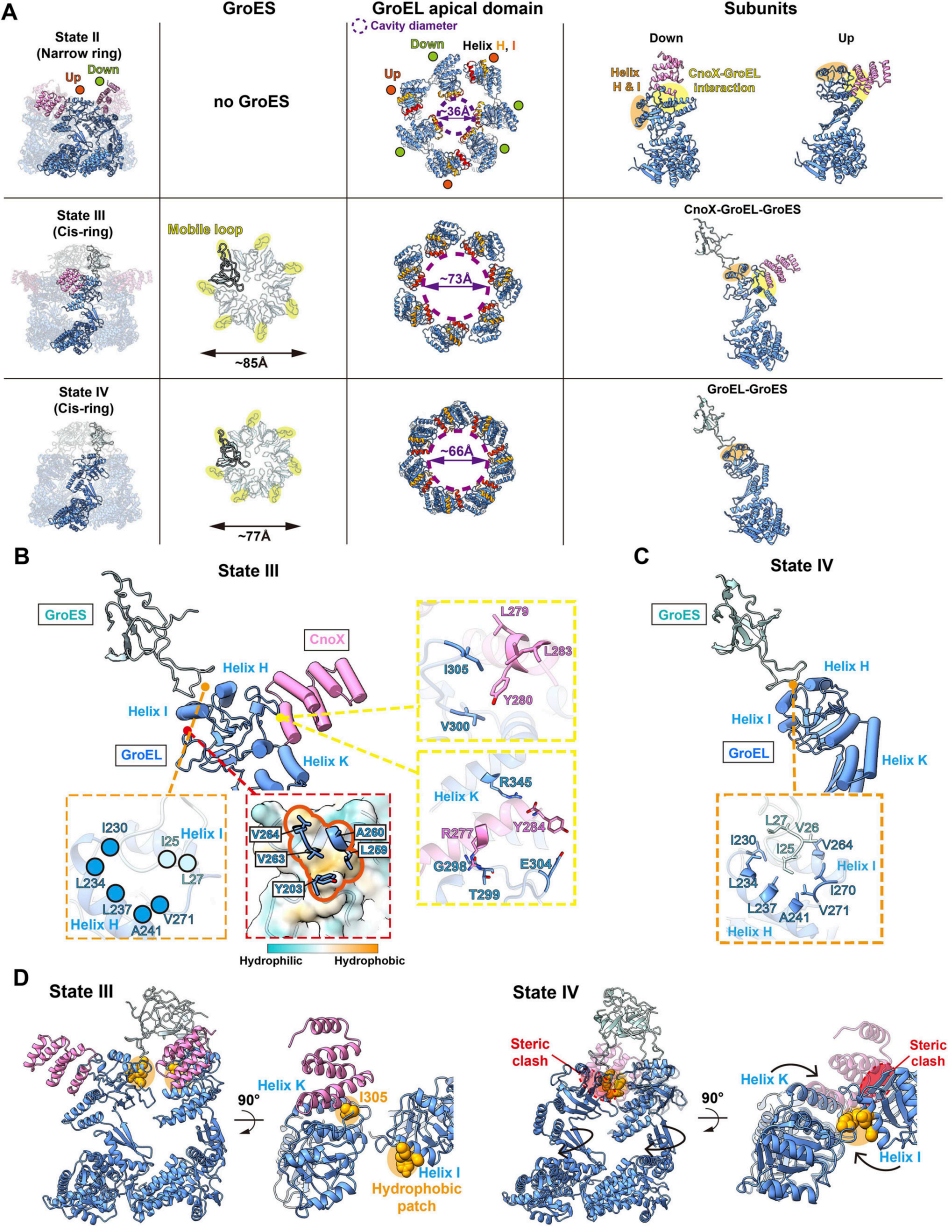
Structural remodeling of the CnoX-binding surface in GroEL/ES assemblies. **(A)** Structural snapshots of GroES, GroEL apical domain, and CnoX–GroEL subunits from state II (narrow ring), state III (cis-ring), and state IV (cis-ring). **(B)** Close-up of the apical domain in the CnoX–GroEL–GroES subunit. CnoX–GroEL interaction is highlighted in yellow with hydrophobic contacts (up) and electrostatic or hydrogen-bonding interactions (down). GroEL–GroES interactions are shown in orange, and the hydrophobic patch involved in apical-domain remodeling is highlighted in red. **(C)** Apical domain of the GroEL–GroES subunit showing the key residues involved in GroES binding. **(D)** Side and top views of states III and IV illustrating remodeling of the CnoX-binding surface. Superposition of the CnoX–GroEL–GroES subunit from state III onto state IV shows that apical-domain compaction and formation of the I305-centered hydrophobic network occlude the groove that accommodates CnoX in state III.

**Figure S6.**
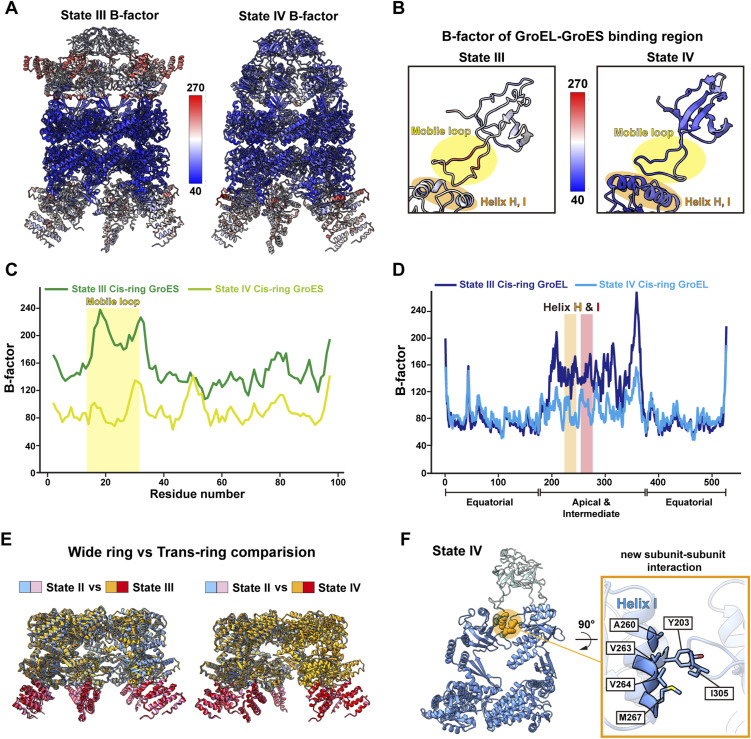
Structural features associated with GroES engagement in states III and IV. **(A)** Atomic models of state III and state IV colored by B-factor on the same scale. **(B)** Close-up of the GroEL–GroES binding interface with its local B-factor colored. **(C)** B-factor profiles comparing GroES between state III and state IV. **(D)** B-factor profiles comparing the cis-ring GroEL subunit between state III and state IV. **(E)** Wide-ring and trans-ring comparisons between states II–III and II–IV. **(F)** Subunit–subunit interaction residues at the newly formed interface of state IV.

Previous biochemical studies have shown that CnoX dissociates from GroEL in the presence of GroES and nucleotides (Dupuy et al, 2023). To define the structural basis of this process, we compared conformational states spanning state II to state IV. In state II, the narrow ring of GroEL exhibits pronounced heterogeneity with “up” and “down” apical conformations (Fig 3A). GroES engagement converts this heterogeneous arrangement into a coordinated upward rotation of all apical domains toward a uniform orientation in state III, repositioning helices H and I toward the GroES mobile loops and establishing an initial docking interface (Fig 3A). This conformational rearrangement expands the apical ring diameter (Fig 3A). In this configuration (state III), GroES binds the cis-ring, whereas CnoX remains attached to both the cis- and trans-rings, demonstrating that the two cofactors can coexist on the same GroEL ring while occupying distinct, non-overlapping apical surfaces (Fig 3A). The GroES density in this state appears weak and shows elevated local B-factors, consistent with a less compact cis-ring conformation (Figs 3A and S6A–D).

State IV reflects a conformation in which GroES is fully engaged with the GroEL ring. Compared with state III, the apical domains of the cis-ring become more compact, leading to ∼7 Å reduction of apical ring diameter (Fig 3A). This conformational tightening converts the flexible, partially docked interface into a more compact chamber in which GroES mobile loops anchor firmly to helices H and I, accompanied by an ∼8° rotation and an ∼8 Å compaction of GroES (Fig 3A). In line with this transition, the overall B-factors of the cis-ring decrease, notably at the GroES and GroEL apical and intermediate domains, reflecting enhanced structural rigidity (Fig S6A–D). Lower B-factors at the GroEL–GroES interface are consistent with a more ordered interaction between the two components (Fig S6B–D). Concomitantly, the additional densities attributable to CnoX vanish from the cis-ring (Fig 1G), suggesting that full GroES engagement is incompatible with accommodation of CnoX on that ring, whereas the trans-ring retains CnoX and remains open (Fig S6E), consistent with the asymmetric GroEL–GroES bullet (Kudryavtseva et al, 2021).

To compare the structural accommodation of CnoX in states III and IV, we analyzed the GroEL apical-domain interfaces. CnoX and GroES occupy distinct binding sites on the GroEL apical domain, enabling simultaneous association without direct competition (Fig 3B). GroES mobile-loop residues form hydrophobic contacts with helices H and I, whereas CnoX engages a separate interface via its C-terminal TPR-B domain (Fig 3B). Compared with state III, state IV exhibits a more extensive GroEL–GroES interface, with each GroEL subunit burying an additional ∼157 Å^2^ of surface area against its corresponding GroES subunit (Fig 3B and C). This expanded interface is accompanied by a more compact arrangement of the GroEL apical domains, which strengthens the hydrophobic network formed by I305 of helix K and neighboring hydrophobic patch residues Y203, L259, A260, V263, and V264 (Figs 3B and D and S6F). These structural rearrangements seal the groove that accommodates CnoX in state III, effectively occluding the CnoX-binding surface in state IV (Fig 3D).

These findings indicate that CnoX and GroES can simultaneously occupy distinct apical surfaces in state III, whereas state IV features a more extensive GroEL–GroES interface, a more compact apical-domain arrangement, and concomitant occlusion of the CnoX-binding groove. Thus, the absence of CnoX density from the cis-ring is consistent with remodeling of the GroEL apical surface rather than direct competition between CnoX and GroES.

### Structural comparison of CnoX-associated and fully GroES-engaged GroEL/ES states

**Figure 4.**
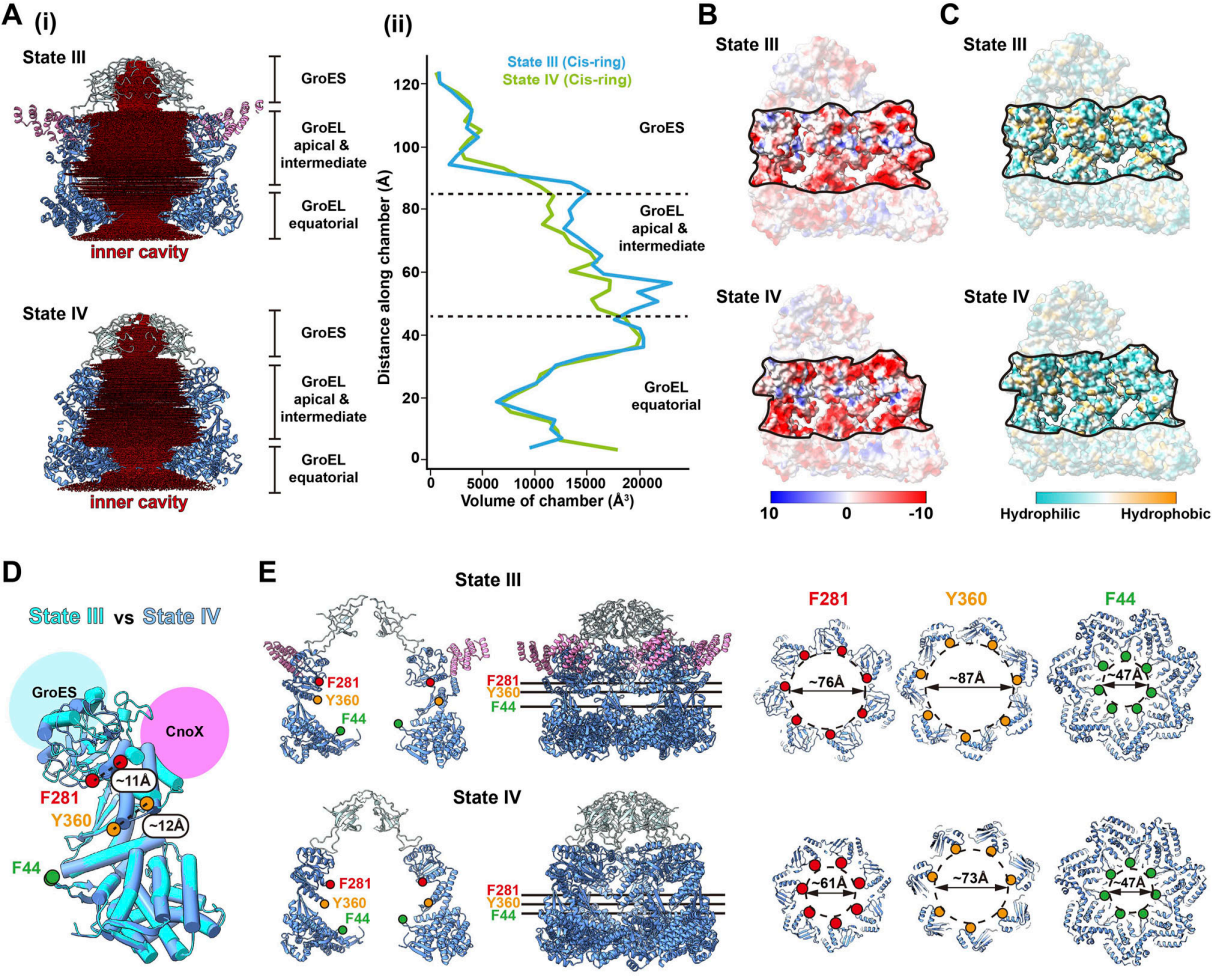
Remodeling of the folding chamber between state III and IV. **(A)** (i) Inner cavities (red mesh) of the state III and state IV cis-rings and (ii) their chamber volume profiles along the chamber axis. **(B)** Electrostatic potential maps of the cis-ring chamber in state III and state IV (blue = positive, red = negative). **(C)** Hydrophobicity maps of the cis-ring chamber in state III and state IV. **(D)** Superposition of GroEL subunits from states III and IV, highlighting movements of F281, Y360, and F44. **(E)** Positions of F281, Y360, and F44 in the cis-rings of states III and IV with measured distances highlighting the relative inward movement of F281 and Y360.

**Figure S7.**
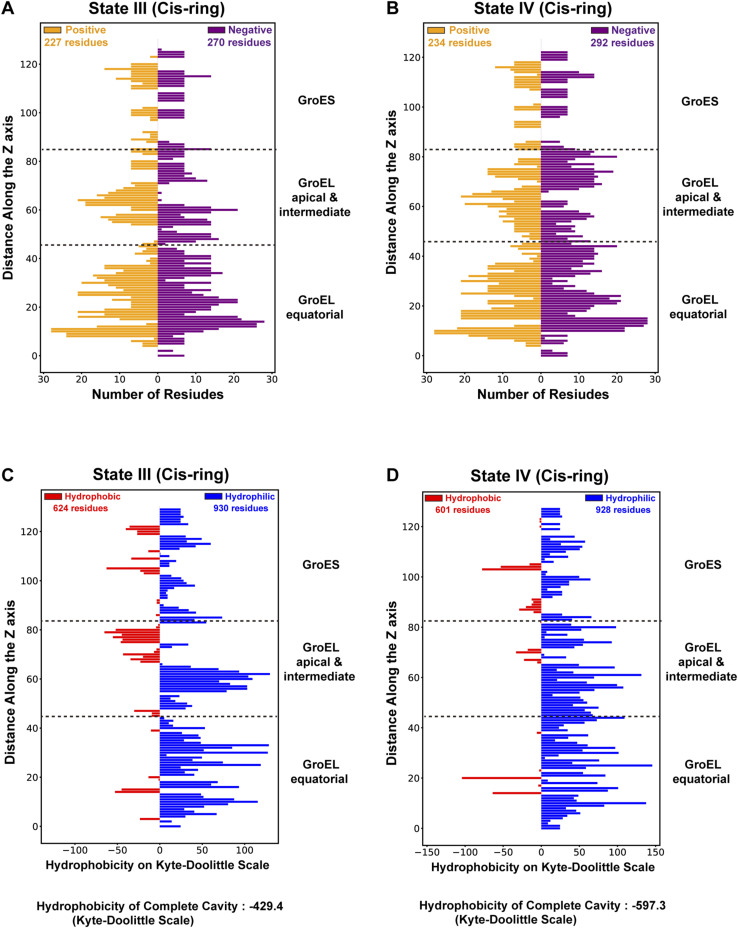
Electrostatic and hydrophobic environment of the GroEL/ES chamber in the presence and absence of CnoX. **(A)** Distribution of positively and negatively charged residues along the z-axis in the chamber of state III cis-ring. **(B)** Distribution of charged residues in the chamber of the state IV cis-ring. **(C)** Distribution of hydrophobic and hydrophilic residues in the chamber of the state III cis-ring. **(D)** Distribution of hydrophobic and hydrophilic residues in the chamber of the state IV cis-ring.

Given that GroEL/ES activity is sensitive to the conformation and physicochemical state of the folding chamber (Tang et al, 2006; England & Pande, 2008; Chakraborty et al, 2010; Clare et al, 2012), we compared the structural characteristics of the cis-ring chambers in state III and state IV. Compared with the state III cis-ring, state IV exhibits an ∼9% reduction in folding-chamber volume (Fig 4A). In addition, electrostatic potential and hydrophobicity analyses reveal that, compared with state III, state IV exhibits an increase in negative net charge and overall hydrophilicity within the chamber (Figs 4B and C and S7A–D). These changes are most prominent in the apical and intermediate domains, which undergo substantial remodeling between state III and state IV (Figs 4A–C and S7A–D). Together, these observations reveal structural differences between the state III and state IV cis-ring chambers, including compaction of the cavity and changes in its physicochemical properties.

Next, we analyzed critical residues known to GroEL/ES activity. F281 and Y360 in the apical domain, and F44 in the equatorial domain, are highly conserved across group I chaperonins and serve as hydrophobic anchors essential for substrate capture and encapsulation (Chen et al, 2013; Kim et al, 2022; Gardner et al, 2023; Braxton et al, 2024; Wagner et al, 2024). The state IV structure exhibits an inward contraction of the apical domains relative to state III (Figs 3A and 4A). Compared with state III, residues F281 and Y360 in state IV are positioned approximately 11 Å and 12 Å further inward, respectively, resulting in an ∼15 Å reduction in inter-residue distance and a tighter folding chamber (Fig 4D and E). In contrast, the equatorial residue F44 shows minimal positional change (Fig 4D and E). Together, these features define the fully GroES-engaged cis-ring architecture in state IV.

Collectively, these results highlight structural differences between the CnoX-associated state III and the fully GroES-engaged state IV GroEL/ES assemblies. These observations establish a structural relationship between CnoX occupancy and GroEL/ES conformational states, providing a framework for understanding how redox co-chaperones are accommodated within chaperonin assemblies.

## Discussion

**Figure 5.**
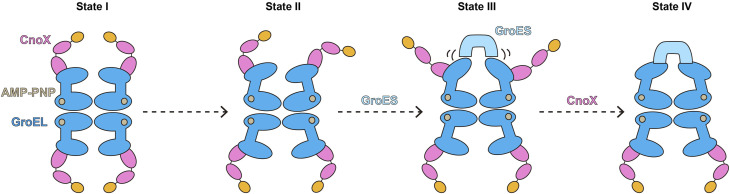
Structural framework for CnoX accommodation across GroEL/ES conformations. Four experimentally observed CnoX-associated GroEL/ES conformations. In state I, CnoX binds symmetrically to both GroEL rings in the presence of AMP-PNP (gray). State II exhibits asymmetric apical-domain rearrangements while maintaining CnoX association through the conserved TPR-B–mediated interface. In state III, GroES (light blue) and CnoX simultaneously occupy the same GroEL ring, demonstrating that their binding sites are structurally distinct and non-overlapping. In state IV, GroES adopts a more compact conformation and the CnoX-binding groove becomes occluded, resulting in the absence of CnoX density from the cis-ring. These states define distinct structural configurations through which CnoX can be accommodated or excluded during remodeling of GroEL/ES assemblies. The relative temporal ordering of these conformations within the physiological GroEL/ES reaction cycle remains unresolved.

In this study, we resolved four structurally distinct nucleotide-bound states of CnoX-associated GroEL/ES assemblies (Fig 1), providing a structural view of how the redox co-chaperone CnoX is accommodated across diverse chaperonin conformations. These structures reveal that CnoX remains associated with GroEL through a conserved TPR-B–mediated interface despite substantial rearrangements of the apical domains (Fig 2). We further captured a ternary GroEL–GroES–CnoX assembly in which CnoX and GroES simultaneously occupy the same GroEL ring, demonstrating that their binding sites are structurally distinct and non-overlapping (Fig 3). Comparison of two GroES-bound states shows that apical-domain compaction remodels the CnoX-binding surface and coincides with the loss of CnoX density from the cis-ring (Fig 3). This structural remodeling is accompanied by changes in folding-chamber architecture, including compaction of the cavity and alterations in its physicochemical properties (Fig 4). Whereas state I resembles the previously reported CnoX–GroEL structure (Dupuy et al, 2023), states II–IV reveal additional CnoX-associated GroEL/ES conformations, including a ternary GroEL–GroES–CnoX assembly and a GroES-bound state in which the CnoX-binding groove becomes structurally occluded. Together, these observations provide a structural framework for understanding how CnoX is accommodated and excluded across distinct GroEL/ES conformations (Fig 5).

Comparison of states III and IV provides insight into structural differences associated with GroES engagement. In state III, GroES density is comparatively weak and local resolution is reduced at the mobile-loop region, indicating structural heterogeneity at the GroEL–GroES interface (Figs S3E and S6A–C). By contrast, state IV exhibits a more compact GroES conformation accompanied by lower B-factors throughout the cis-ring and at the GroEL–GroES interface, consistent with increased ordering of both GroES and the surrounding apical domains (Figs 3A–C and S6A–D). These observations are consistent with previous biochemical and structural studies showing that the GroES mobile loop undergoes a disorder-to-order transition upon productive interaction with GroEL and contributes to stabilization of the GroEL–GroES complex (Richardson et al, 1999; Shewmaker et al, 2004). Notably, the same apical-domain remodeling that accompanies increased ordering of the GroEL–GroES interface also occludes the CnoX-binding groove (Fig 3D). Thus, our structures indicate that increased ordering of the GroEL–GroES interface is accompanied by occlusion of the CnoX-binding groove in the fully GroES-engaged architecture.

Although the structural relationships among the observed conformations are well defined, their relationship to the physiological GroEL/ES reaction cycle remains unresolved. Future studies incorporating additional nucleotide states, substrate-bound complexes, and time-resolved structural approaches will be required to determine how these conformations relate to chaperonin function in vivo. Nevertheless, the structures presented here establish a framework for understanding how a redox co-chaperone can be accommodated and excluded during remodeling of GroEL/ES assemblies and provide a basis for future studies investigating the relationship between redox quality control and chaperonin-mediated protein folding (Fig 5).

## Materials and Methods

### Protein expression and purification

GroEL: pET22b, containing the GroEL sequence (UniProt ID: P0A6F5), was transformed into *E. coli* BL21 (DE3). Cells were grown at 37°C until an OD600 of 0.6 was reached. Protein expression was induced with 0.5 mM isopropyl β-D-thiogalactopyranoside (IPTG) for 4 h. After centrifugation, cell pellets were resuspended in buffer A (50 mM Tris–HCl, pH 7.4, 1 mM EDTA, and 1 mM DTT) and lysed by sonication. Lysates were clarified by centrifugation at 27000*g* for 30 min. The supernatant was filtered through a 0.45-μm membrane. The cleared lysate was loaded onto a 5-ml HiTrap Q HP column using buffer A and buffer B (50 mM Tris–HCl, pH 7.4, 1 M NaCl, 1 mM EDTA, and 1 mM DTT). GroEL was eluted with 300–400 mM NaCl; we concentrated the protein-containing solution using Amicon Ultra 50 kDa MWCO concentrators. After concentration, we purified the sample by gel filtration chromatography using a Superose 6 16/600 column and buffer C (50 mM Tris–HCl, pH 7.4, 10 mM MgCl_2_, and 50 mM KCl). After purification, we performed the same procedure (anion exchange chromatography using a Hitrap Q HP column and gel filtration chromatography using a Superose 6 10/300 column) one more time. The final protein was concentrated, aliquoted, snap frozen, and stored at −80°C.

GroES: pET22a, containing the GroES sequence (UniProt ID: P0A6F9), was transformed into *E. coli* BL21 (DE3). Cells were grown at 37°C until an OD600 of 0.6 was reached. We then applied 0.5 mM isopropyl β-D-thiogalactopyranoside (IPTG) for 4 h. After centrifugation, cells were resuspended in buffer A and lysed by sonication. Centrifugation was performed at 27000*g* for 30 min. A 0.45-μm filter was used to filter the supernatant. The cleared lysate was loaded onto a 5-ml HiTrap Q HP column using buffer A and buffer B. GroES was eluted at 130–250 mM NaCl. Protein-containing fractions were concentrated using Amicon Ultra 10 kDa MWCO concentrators. Concentrated protein was further purified by size-exclusion chromatography on a Superdex 200 10/300 column equilibrated with buffer D (50 mM Tris–HCl, pH 7.4 and 100 mM NaCl). After purification, the fractions were concentrated and heated at 60°C for 10 min. The precipitate was removed, and gel filtration chromatography was conducted again with the supernatant. The final protein was concentrated, aliquoted, snap frozen, and stored at −80°C.

CnoX: pET28a, containing the CnoX sequence (UniProt ID: P77395), was transformed into *E. coli* BL21 (DE3) cells. CnoX was expressed with an N-terminal 6xHis-tag to facilitate purification and maintain GroEL interaction. Cells were grown at 37°C to an OD600 of 0.6 and induced with 0.5 mM IPTG for 4 h. Cells were harvested, resuspended in buffer A, and lysed by sonication. Lysates were clarified by centrifugation (27000*g*, 30 min) and filtration through a 0.45-μm filter. The cleared lysate was loaded onto a 5-ml HisTrap HP column equilibrated with buffer E (50 mM Tris–HCl, pH 7.4, 200 mM NaCl, and 20 mM imidazole) and eluted with buffer F (50 mM Tris–HCl, pH 7.4, 200 mM NaCl, and 300 mM imidazole). Protein-containing fractions were concentrated using Amicon Ultra 10 kDa MWCO concentrators. Concentrated protein was further purified by size-exclusion chromatography on a Superdex 75 10/300 column equilibrated with buffer D. The final protein was concentrated, aliquoted, snap frozen, and stored at −80°C.

### Cryo-EM specimen preparation and data collection

CnoX and GroEL with AMP-PNP: 10 μM GroEL was used for sample preparation. GroEL and CnoX were incubated at room temperature in a 1:14 molar ratio (14[GroEL14] = [CnoX]) for 15 min. Then 1 mM AMP-PNP, 10 mM MgCl_2_, and 50 mM KCl were added, and the mixture was incubated for 30 min. 3 μl of the sample was applied to glow-discharged 200-mesh R1.2/1.3 holey-carbon grids (Quantifoil), blotted for 3.5 s, and plunge-frozen in liquid ethane. All procedures were performed using a Vitrobot Mark IV (Thermo Fisher Scientific). Data were collected on a Glacios (Thermo Fisher Scientific) equipped with a Falcon 4 detector, yielding 2,435 movies.

CnoX, GroEL, and GroES with AMP-PNP: 10 μM GroEL was used for sample preparation. GroEL and CnoX were incubated at room temperature at a 1:14 molar ratio (14[GroEL_14_] = [CnoX]) for 15 min. Then, purified GroES was mixed with GroEL at a 1:2 stoichiometry, followed by the addition of 5 mM AMP-PNP, 10 mM MgCl_2_, and 50 mM KCl. The sample was then incubated for 40 min. Before grid application, 0.05% n-octyl-β-D-glucopyranoside was added to improve particle orientation distribution. An aliquot of 3 μl was applied to glow-discharged 200-mesh R1.2/1.3 holey-carbon grids (Quantifoil), blotted for 3.5 s, and plunge-frozen in liquid ethane using a Vitrobot Mark IV (Thermo Fisher Scientific). A total of 3,075 movies were collected on a Glacios (Thermo Fisher Scientific) equipped with a Falcon 4 detector.

### Data processing and 3D refinement

All image processing was performed in cryoSPARC v4 (Punjani et al, 2017).

State I and state II: Using patch motion correction and patch CTF estimation in cryoSPARC (Punjani et al, 2017), movies were aligned and the CTF estimated. Template-based autopicking with subsequent 2D classification yielded 353,962 selected particles. After *ab initio* reconstruction and homogenous refinement (C1), state I and state II complexes were distinguished by 3D classification. A total of 214,141 particles were classified as state I. Non-uniform refinement (Punjani et al, 2020) with no symmetry and with C7 symmetry imposed yielded maps at 3.11 Å and 2.73 Å, respectively. The map was sharpened using DeepEMhancer (Sanchez-Garcia et al, 2021). 51,352 particles are obtained for state II, and by using homogenous refinement, we obtained a 3.35 Å consensus map. To obtain a clear CnoX TPR B density, we attempted to impose a C7 symmetry. However, the TPR B domain in the asymmetric ring was obscure. To relax the symmetry of the asymmetric ring, a soft mask was applied to the heterogeneous ring. Using symmetry expansion (C7) and 3D variability analysis (Punjani & Fleet, 2021), 64,559 well-resolved CnoX particles were classified. Duplicate particles were removed, yielding 38,808 particles. Using these particles, we performed non-uniform refinement (Punjani et al, 2020) in C1 symmetry and local refinement in C1 symmetry with a mask for a heterogeneous ring. Because one subunit had an unclear structure, we conducted focused 3D classification using a soft mask. Based on the 3D classification, 29,790 particles were classified. We used C1 non-uniform refinement (Punjani et al, 2020) and C1 local refinement using a mask on one ring. We obtained 3.46 Å state II map from refinement and sharpened the map using DeepEMhancer (Sanchez-Garcia et al, 2021). A summary illustration of the image processing is shown in Fig S2B.

State III and state IV: To align movies and estimate their CTF, we applied patch motion correction and patch CTF estimation using cryoSPARC (Punjani et al, 2017). Autopicking based on the template and 2D classification was performed to select 445,122 suitable particles. Ab initio and heterogeneous refinement yielded 143,513 state III and 133,737 state IV particles. For state III, we conducted homogenous refinement in C7 symmetry imposed and obtained a 2.84 Å consensus map. After refinement, to obtain a clear density of CnoX using a mask on the CnoX–GroEL–GroES ring, we performed local refinement with C7 symmetry imposed. We obtained a 2.83 Å final map. We used the DeepEMhancer (Sanchez-Garcia et al, 2021) for map sharpening. We also tested homogenous refinement with no symmetry but obtained a similar map with low resolution (Fig S3F). Therefore, for a high resolution, we used a C7 imposed map. For state IV, we also conducted homogenous refinement with C7 symmetry imposed. After refinement, we performed non-uniform refinement (Punjani et al, 2020) with C7 symmetry imposed, obtained a 2.79 Å final map, and sharpened map with DeepEMhancer (Sanchez-Garcia et al, 2021). A summary illustration of the image processing is shown in Fig S3B.

### Model building

State I: The previously reported cryo-EM structure of CnoX–GroEL (PDB 7YWY [Dupuy et al, 2023]) was used as a reference. A C7-symmetry–imposed map at 2.73 Å resolution was used for model building. We used UCSF Chimera v1.8 (Pettersen et al, 2021) for rigid body fitting in the map and ISOLDE v1.8 (Croll, 2018) for initial refinement. After the initial refinement, the model was manually refined in COOT (Emsley & Cowtan, 2004) using Phenix.real_space_refine (Afonine et al, 2018) for several rounds.

State II: The previous GroEL model (PDB 8BA8 [Gardner et al, 2023]) was used as a reference for GroEL, and the crystal structure of CnoX (PDB 3QOU [Lin & Wilson, 2011]) was used as a reference for CnoX. Using UCSF Chimera v1.8 (Pettersen et al, 2021), we fitted the structures to the map, used ISOLDE v1.8 (Croll, 2018), performed initial refinement, and manually refined the initial model using COOT (Emsley & Cowtan, 2004). We then used Phenix.real_space_refine (Afonine et al, 2018) for the final refinement.

State III: The previously reported crystal structure of GroEL–GroES (PDB 1AON [Xu et al, 1997]) was used as a reference. The crystal structure of CnoX (PDB: 3QOU [Lin & Wilson, 2011]) was used as a reference. Chimera v1.8 (Pettersen et al, 2021) was used for the rigid body fitting of the models to the map. Isolde v1.8 (Croll, 2018) was used for initial refinement. COOT (Emsley & Cowtan, 2004) was used for manual refinement of the model and Phenix.real_space_refine (Afonine et al, 2018) was used for final refinement.

State IV: Previous crystal structures of GroEL–GroES (PDB 1AON [Xu et al, 1997]) and CnoX (PDB 3QOU [Lin & Wilson, 2011]) were used as references. UCSF Chimera v1.8 (Pettersen et al, 2021) and ISOLDE v1.8 (Croll, 2018) were used for rigid-body fitting of the map and initial refinement. We used COOT (Emsley & Cowtan, 2004) for manual refinement and Phenix.real_space_refine (Afonine et al, 2018) for the final refinement.

### Structural analysis

Domain movement angles were analyzed using DynDom 6D (Veevers & Hayward, 2019). Electrostatic potential, hydrophobicity analyses, and cavity volume measurements of state III and state IV cis-rings were done by CICLOP (Garg et al, 2022). All other structural features of models were analyzed using UCSF ChimeraX v1.8 (Pettersen et al, 2021) and UCSF Chimera (Pettersen et al, 2004).

## Supplementary Material

Reviewer comments

## Data Availability

Cryo-EM maps used for the studies have been deposited in EMDB (Electron Microscopy Data Bank). Atomic coordinates for the cryo-EM density maps have been deposited in PDB (Protein Data Bank). For state I consensus, the map is available in EMDB-65953. For state I, the map is available in EMDB-65877 and the model is available in PDB 9WCW. For state II, the map is available in EMDB-65990 and the model is available in PDB 9WID. For state III, the map is available in EMDB-65992 and the model is available in PDB 9WIF. For state IV, the map is available in EMDB-65991 and the model is available in PDB 9WIE.
