# Supplementary material for: Structural interplay of the redox co-chaperone CnoX to GroEL/ES chaperonin
Source: Life Sci Alliance. 2026 Aug 7;9(10):e202603764. doi: 10.26508/lsa.202603764 (PMC13451151; doi:10.26508/lsa.202603764)
Supplement: Supplementary file 1 [file LSA-2026-03764_review_history.pdf]

Structural interplay of the redox co-chaperone CnoX to GroEL/ES chaperonin

Junhak Kim, Mingyu Jung, and Soung-Hun Roh
DOI: https://doi.org/10.26508/lsa.202603764

Corresponding author(s): Soung-Hun Roh, Seoul National University

**Review Timeline:**		
	Submission Date:	2026-05-11
	Editorial Decision:	2026-05-14
	Revision Received:	2026-06-17
	Editorial Decision:	2026-07-13
	Revision Received:	2026-07-22
	Accepted:	2026-07-24

Scientific Editor: Tim Fessenden

Transaction Report:

(Note: With the exception of the correction of typographical or spelling errors that could be a source of ambiguity, letters and reports are not edited. The original formatting of letters and referee reports may not be reflected in this compilation.)

Please note that the manuscript was previously reviewed at another journal and the reports were taken into account in the decision-making process at *Life Science Alliance*.

May 14, 2026

Re: Life Science Alliance manuscript #LSA-2026-03764-T

Prof. Soung-Hun Roh
Seoul National University
School of Biological Science
1 Gwanakro
105-102
Seoul 08826
Korea, Republic of

Dear Dr. Roh,

Thank you for transferring your manuscript entitled "Structural interplay of the redox co-chaperone CnoX to GroEL/ES chaperonin" to Life Science Alliance. In accordance with our offer to consider this work, and as confirmed in your recent correspondence, we invite you to submit a revised manuscript with the following changes in place:

- Adjust the interpretation and claims throughout the text to reduce emphasis on kinetics and sequential intermediate states and reframe these as speculative, in particular without apo or ADP structures (Reviewers 2 and 3).
- Adjust or remove claims on the proposed effect of CnoX on the ATPase activity of GroEL (Reviewers 2 and 3).
- Address the question from Reviewer 1, with text changes or with new analysis, on the proposed GroES mobile-loop conformational change. Conversely temper the claims on this loop per Point 2 by Reviewer 2.

You are welcome to implement additional changes suggested by the reviewers to improve your manuscript in the manner of your choosing.

To upload the revised version of your manuscript, please log in to your account: https://lsa.msubmit.net/cgi-bin/main.plex

You will be guided to complete the submission of your revised manuscript and to fill in all necessary information. Please get in touch in case you do not know or remember your login name.

While you are revising your manuscript, please also attend to the below editorial points to help expedite the publication of your manuscript. Please direct any editorial questions to the journal office. When submitting the revision, please include a letter addressing the reviewers' comments point by point.

Thank you for this interesting contribution to Life Science Alliance. We are looking forward to receiving your revised manuscript.

Sincerely,

Tim Fessenden
Scientific Editor
Life Science Alliance

A. THESE ITEMS ARE REQUIRED FOR REVISIONS

-- A letter addressing the reviewers' comments point by point.

-- An editable version of the final text (.DOC or .DOCX) is needed for copyediting (no PDFs).

-- High-resolution figure, supplementary figure and video files uploaded as individual files: See our detailed guidelines for preparing your production-ready images, https://www.life-science-alliance.org/authors

-- Summary blurb (enter in submission system): A short text summarizing in a single sentence the study (max. 200 characters including spaces). This text is used in conjunction with the titles of papers, hence should be informative and complementary to the title and running title. It should describe the context and significance of the findings for a general readership; it should be written in the present tense and refer to the work in the third person. Author names should not be mentioned.

-- By submitting a revision, you attest that you are aware of our payment policies found here: https://www.life-science-alliance.org/copyright-license-fee

B. MANUSCRIPT ORGANIZATION AND FORMATTING:

Full guidelines are available on our Instructions for Authors page, https://www.life-science-alliance.org/authors

We encourage our authors to provide original source data, particularly uncropped/-processed electrophoretic blots and spreadsheets for the main figures of the manuscript. If you would like to add source data, we would welcome one PDF/Excel-file per figure for this information. These files will be linked as supplementary "Source Data" files.

IMPORTANT: It is Life Science Alliance policy that if requested, original data images must be made available. Failure to provide original images upon request will result in unavoidable delays in publication. Please ensure that you have access to all original microscopy and blot data images before submitting your revision.

Kim et al, Response to Reviewer Comments :
Response to Reviewer #1

Kim, Jung, and Roh present a series of cryo-EM structures of GroEL/CnoX and GroEL/GroES/CnoX complexes and ATPase activity measurements of the protein mixtures. The major finding is the appearance of a new conformational state (State III) of GroEL-GroES that is stabilized by binding of the CnoX redox chaperone. Analysis of State III reveals partial narrowing of the GroEL ring toward the established GroEL14-ATP7-GroES7 state (State IV) and the concomitant intermediate-domain and apical-domain rotations. CnoX stabilization of this state is described as a checkpoint that delays the completion of the canonical GroEL-ATP7-GroES folding chamber. The somewhat reduced ATPase activity in GroEL/GroES/CnoX, compared to GroEL/GroES, supports this conclusion. The speculation that the delayed transition permits CnoX redox activity to act on the unfolded client is attractive but remains an open question. The gradual rotation of CnoX from pointing into the GroEL-wide chamber to access the unfolded client, then pointing up as the GroEL ring becomes more narrow, then outward as GroES binds is also attractive but very speculative.
Importantly, the new GroEL/GroES/CnoX structures demonstrate that CnoX is not displaced by GroES binding (as suggested in others' work), but rather that it is discharged by a GroEL conformational change that is completed by the State III>IV shift. This conclusion is supported by the mapping of respective interfaces and identifying side-chain packing that occludes the CnoX-binding site.

We thank the reviewer for the positive assessment of our structural data and for recognizing the importance of the newly identified GroEL–GroES–CnoX ternary assembly.

The story is simple and nearly complete but for the lack of analysis of the GroES mobile-loop conformational change. This would appear to be critical because GroES binding is described as driving a GroEL conformational change (Fig. 3, legend). Differences in the mobile loop can be seen when comparing Fig. 3C and 3D, but they are not described. Is 3D more stable, such that it could "drive" the III-IV shift and discharge CnoX? Earlier work of Georgopoulos and coworkers showed that single-amino-acid changes in the mobile loop could compensate for mutations in GroEL that affect its conformation changes. Is one or other of the mobile loop conformations more stable, and is it consistent with the earlier studies?

We thank the reviewer for this insightful comment. To address this point, we performed additional analyses of local resolution and B-factor distributions at the GroEL–GroES interface and incorporated the results into the revised Results and Discussion sections.

In State III, the GroES mobile-loop region is poorly resolved, with a local resolution of approximately 5–8 Å, indicating substantial conformational heterogeneity. Therefore, we cannot assign a unique atomic conformation to the mobile loop in this state, and its interaction with GroEL is interpreted from the density envelope and previously established GroEL–GroES interfaces. In contrast, State IV shows improved local resolution and lower B-factors at the

GroEL–GroES interface, indicating that the mobile-loop region and the surrounding GroEL apical domains are more ordered in State IV.

We emphasize, however, that improved local resolution and reduced B-factors indicate increased structural ordering rather than directly demonstrating greater thermodynamic stability. Our data also do not establish that mobile-loop ordering drives the State III-to-IV transition or directly causes CnoX dissociation. Rather, the increased ordering of the GroEL–GroES interface in State IV occurs concurrently with remodeling of the GroEL apical domains and occlusion of the CnoX-binding site.

This interpretation is consistent with earlier genetic studies from Georgopoulos and colleagues showing that substitutions in the GroES mobile loop can compensate for GroEL mutations affecting its conformational cycle. These studies support a functional coupling between mobile-loop interactions and GroEL allosteric transitions. Our structural observations are compatible with such coupling, but they do not allow us to determine whether mobile-loop rearrangement is the primary driver of the III-to-IV transition.

Response to Reviewer #2

The authors used single-particle cryo-electron microscopy to determine four distinct conformational states of the GroEL/ES complex in association with the chaperedoxin CnoX. Across all four states, only the TPR-B domain of CnoX is resolved and observed to interact with the apical domain of GroEL, whereas the Trx and TPR-A domains remain unresolved in the electron density maps. The structures were derived from two cryo-EM datasets: one containing GroEL-CnoX and another containing GroEL-CnoX-GroES all in the presence of the non-hydrolyzable ATP analogue AMP-PNP, demonstrating that a ternary complex can form.

States I and II correspond to CnoX-GroEL assemblies formed in the absence of GroES. State I adopts a nearly symmetric, apo-like architecture, whereas state II displays pronounced inter-ring asymmetry, with the two rings assuming distinct "narrow" and "wide" conformations.

States III and IV represent ternary assemblies of CnoX-GroEL-GroES. In state III, GroES engages the cis-ring while CnoX remains bound to both the cis- and trans-rings. State IV exhibits the canonical bullet-shaped GroEL-GroES architecture, characterized by strong GroES density on the cis-ring and the absence of detectable CnoX density on that ring, while CnoX remains associated with all seven subunits of the trans-ring.

One of the conformations (state I) resembles a previously reported structure (Dupuy et al., 2023), while the remaining three represent novel conformational states.

Based on these structural snapshots, the authors propose a mechanistic model describing how CnoX participates in the GroEL/ES chaperoning cycle. Functionally, the study is limited to ATPase assays of GroEL in the presence of GroES, CnoX, or both.

While the structural data are of high quality, the proposed mechanistic model remains speculative and would benefit from additional functional validation.

We thank the reviewer for the careful evaluation of our work and for the constructive suggestions.

The authors describe ATP-driven transitions between state I and state II (Fig. 2F). However, both conformations were obtained from the same cryo-EM dataset in the presence of AMP-PNP. Since AMP-PNP is non-hydrolyzable, it is unclear how ATP-driven transitions can be inferred.

We agree with the reviewer. Throughout the revised manuscript, we have removed ATP-driven and sequential interpretations of the observed conformations. States I–IV are now presented as distinct nucleotide-bound structural states observed under AMP-PNP conditions. Figures 2 and 5 have been redesigned accordingly, and all references to ordered progression through the GroEL/ES cycle have been removed or explicitly stated as unresolved.

In state III, the GroES mobile loop is modeled to interact with helices H and I of GroEL, yet this region is not clearly resolved in the density.

We agree and have substantially revised our interpretation. We now explicitly state the limited local resolution of the mobile-loop region and have tempered our conclusions accordingly. Additional local-resolution and B-factor analyses were incorporated to provide a more conservative interpretation of the GroEL–GroES interface.

The authors propose that state III represents an intermediate where both CnoX and GroES are bound to GroEL cis-ring, preceding full GroES engagement and CnoX displacement (state IV), and that the loss of CnoX density of the cis-ring arises not from direct competition with GroES but from allosteric restructuring of GroEL itself.

- How stable is this intermediate state?
- Can the ternary complex (state III) be isolated in solution (e.g., by SEC-MALS)?
- Or is this state transient and primarily observable under cryo-EM conditions?
- From the cryo-EM data processing (Fig. S3), state III represents ~32% of particles. Does this reflect its population in solution?
- What is the affinity of CnoX for GroEL alone versus GroEL/ES?
- Does GroES binding modulate CnoX affinity?

We agree that such experiments would further strengthen the study. However, because the present work focuses on structural characterization, we have limited our conclusions to observations directly supported by the cryo-EM data. Accordingly, statements regarding stability, affinity, kinetic behavior, and temporal ordering have been removed from the manuscript.

The authors assign low-contour density within the GroEL cavity to the Trx and TPR-A domains of CnoX (Fig. 2E). The conclusions regarding positioning of these domains inside the folding chamber appear overinterpreted and should be presented more cautiously.

We appreciate this concern and have substantially tempered our interpretation. These densities are now described as low-confidence assignments that may reflect flexible regions of CnoX. We no longer draw mechanistic conclusions from these features.

Is CnoX a general component of the GroEL/ES machinery or acts selectively. Is CnoX required for all GroEL substrates, or specifically for cysteine-containing proteins requiring redox regulation?

We agree that this is an important biological question. Because the current study focuses on structural characterization, we have revised the Discussion to avoid broader functional claims and instead present these questions as important directions for future investigation.

The authors report that CnoX inhibits the ATPase activity of the GroEL/GroES complex; however:

• Structurally, it is unclear how CnoX exerts this effect.
• Unlike GroES, which directly stabilizes the apical domain and restricts conformational cycling, CnoX binding appears more peripheral.
• CnoX alone does not significantly affect GroEL ATPase activity.
• What was the order of protein mixing in the ATPase assay?
• If GroEL/ES is already formed, can CnoX still associate?
• Can the complex revert to a state that accommodates CnoX during ATPase cycling?

This discrepancy should be addressed mechanistically. This has direct implications for interpreting the ATPase assay results.

We agree with the reviewer. All ATPase data, analyses, and associated interpretations have been removed from the revised manuscript.

The study lacks structures in the presence of client proteins.

We agree that client-bound structures would be highly informative. We now discuss this as an important direction for future studies but do not make conclusions regarding substrate engagement based on the current data.

In the final model (Fig. 5), none of the four experimentally determined states are directly represented. Instead, schematic single- and double-capped GroEL/ES complexes with CnoX are shown.

• The model does not clearly map onto the four described states.
• Substrate engagement is included despite not being structurally or functionally characterized.
• Nucleotide transitions (ATP → ADP) are not clear in the proposed final model.

We substantially redesigned Figure 5. The revised figure now focuses on the experimentally observed structural states and their structural relationships. Mechanistic and kinetic

interpretations have been removed, and the figure no longer implies a defined progression through the GroEL/ES reaction cycle.

Response to Reviewer #3

Previous work by Collet and co-workers (Cell 2023) characterized the interaction of CnoX, a protein that combines redox and chaperone functions, with GroEL. This 2023 Cell paper includes a cryo-EM structure of CnoX bound to GroEL and functional analysis. The paper considered here extends this work by providing structures of three additional states: CnoX in complex with GroEL bound to an ATP analog and CnoX bound to either the trans or to both GroEL rings in the GroEL/ES complex. A revision should address the points below:

We thank the reviewer for the constructive comments and for highlighting the limitations of the ATPase experiments and kinetic interpretations.

1. The authors make claims about the effects of CnoX on the ATPase activity of GroEL based on a single time-point of 30 min using 100 uM ATP and 125 nM GroEL. Under these conditions, most of the ATP is hydrolyzed by 30 min, and there is no steady state. Comparisons between different conditions (e.g. with or without CnoX, GroES etc.) are meaningless unless done under steady state conditions.
2. The claim that CnoX has no effect on the ATPase activity of GroEL also ignores potential effects of CnoX on cooperativity in the ATPase activity of GroEL. Observing such an effect requires measuring initial rates of ATPase activity of GroEL at many fixed concentrations of ATP, which has not been done.
3. The authors make many claims about the effects of CnoX on the kinetics of the GroEL machine. For example, it is stated that: 'CnoX transiently delays GroEL compaction and ATP-driven cycling', 'CnoX does not perturb GroEL's intrinsic conformational dynamics' and that CnoX acts as a 'redox dependent kinetic modulator'. However, no kinetic experiments are reported, and all these claims are, therefore, speculations based on observations of static structures.

We agree with the reviewer. Because the ATPase measurements were insufficient to support mechanistic conclusions regarding GroEL ATPase regulation, we have removed all ATPase data, analyses, and associated interpretations from the revised manuscript. In addition, all statements regarding kinetic modulation, delayed cycling, kinetic checkpoints, or ATPase regulation by CnoX have been removed.

4. The authors ignore potential artefacts due to using AMP-PNP as an ATP analog. For example, it has been reported that AMP-PNP does not induce allosteric transitions in GroEL (Biochim Biophys Acta. 2001 Feb 9;1545(1-2):160-73).

We agree and have revised the manuscript accordingly. We now explicitly acknowledge that all structures were determined under AMP-PNP conditions and refrain from assigning ATP-driven transitions or directional progression among the observed conformations. States I–IV are now presented as distinct nucleotide-bound structural states rather than sequential intermediates.

5. Fig. 4a(ii) is not clear. Also, 'chamber' is mis-spelled.

We thank the reviewer for identifying these issues. The figure presentation has been revised and typographical errors have been corrected.

July 13, 2026

RE: Life Science Alliance Manuscript #LSA-2026-03764-TR

Prof. Soung-Hun Roh
Seoul National University
School of Biological Science
1 Gwanakro
105-102
Seoul 08826
Korea, Republic of (South Korea)

Dear Dr. Roh,

Thank you for submitting your revised manuscript entitled "Structural interplay of the redox co-chaperone CnoX to GroEL/ES chaperonin". We returned this work to Reviewers 1 and 2, whose reports are below.

As you will see, both reviewers are now satisfied with no further major requests. We invite you to consider their suggestions, related to the references (Reviewer 1) and structural ordering vs thermodynamic stability in the mobile-loop of GroES (Reviewer 2). We would be happy to publish your paper in Life Science Alliance pending these and final revisions necessary to meet our formatting guidelines.

MANUSCRIPT ORGANIZATION AND FORMATTING:

To avoid unnecessary delays in the acceptance and publication of your paper, please read the following information carefully. Full guidelines are available on our Instructions for Authors page, https://www.life-science-alliance.org/authors

Submission of a paper that does not conform to Life Science Alliance guidelines will delay the acceptance of your manuscript.

-Please add the X and Bluesky handles of your host institute/organization, as well as your own, and/or one of the authors, in our system.
-Please use the [10 author names et al.] format in your references (i.e., limit the author names to the first 10).
-Please add callouts for Figures S2A, S2C-G; S3A; S4C-H; S5A-B and S7A-D to your main manuscript text.
-Please indicate scale bar sizes in the legends for figures S2A and S3A.

We encourage our authors to provide original source data, particularly uncropped/-processed electrophoretic blots and spreadsheets for the main figures of the manuscript. If you would like to add source data, we would welcome one PDF/Excel-file per figure for this information. These files will be linked as supplementary "Source Data" files.

It is Life Science Alliance policy that if requested, original data images must be made available to the editors. Failure to provide original images upon request will result in unavoidable delays in publication. Please ensure that you have access to all original data images prior to final submission.

If you are planning a press release on your work, please inform us immediately to allow informing our production team and scheduling a release date.

We welcome submissions of potential cover images for the issue of LSA in which your work would appear. If you have high quality images associated with this work, please feel free to email these, with a caption, to the journal office.

LSA encourages authors to provide a 30-60 second video where the study is briefly explained. These videos will be appear embedded with the manuscript online at Life Science Alliance, and on social media to promote the published paper and authors (for examples, see https://docs.google.com/document/d/1-UWCfbE4pGcDdcgzcmiuJl2XMBJnxKYeqRvLLrLSo8s/edit?usp=sharing). Corresponding or first-authors are welcome to submit the video. Please submit only one video per manuscript. The video can be emailed to contact@life-science-alliance.org

FINAL FILES:

To upload the final version of your manuscript, please log in to your account: https://lsa.msubmit.net/cgi-bin/main.plex
You will be guided to complete the submission of your revised manuscript and to fill in all necessary information. Please get in touch in case you do not know or remember your login name.

The following items are required for acceptance.

-- An editable version of the final text (.DOC or .DOCX) is needed for copyediting (no PDFs).

-- High-resolution figure, supplementary figure and video files uploaded as individual files: See our detailed guidelines for preparing your production-ready images, https://www.life-science-alliance.org/authors

-- Summary blurb (enter in submission system): A short text summarizing in a single sentence the study (max. 200 characters including spaces). This text is used in conjunction with the titles of papers, hence should be informative and complementary to the title. It should describe the context and significance of the findings for a general readership; it should be written in the present tense and refer to the work in the third person. Author names should not be mentioned.

The license to publish form must be signed before your manuscript can be sent to production. A link to the license to publish form will be available to the corresponding author only. Please take a moment to check your funder requirements.

Reviews, decision letters, and point-by-point responses associated with peer-review at Life Science Alliance will be published, alongside the manuscript. If you do want to opt out of having the reviewer reports and your point-by-point responses displayed, please let us know immediately.

Thank you for your attention to these final processing requirements. Please revise and format the manuscript and upload materials as soon as you are able.

Thank you for this interesting contribution to the literature. We look forward to publishing your paper in Life Science Alliance.

Sincerely,

Tim Fessenden
Scientific Editor
Life Science Alliance

Reviewer #1 (Comments to the Authors (Required)):

This paper describes the structures of four states of CnoX in complex with GroEL bound to an ATP analog and with or without GroES. One state has been described before but the others are new and informative. The revised version is much improved after various speculations and ATPase data have been removed. My only very minor comment is that most of the cited reviews in the paper are old (2001, 2011, 2016) and much has changed. I suggest adding/replacing with newer reviews.

Reviewer #2 (Comments to the Authors (Required)):

Kim et al. report new conformational states of GroEL formed in complex with redox enzyme CnoX and GroES. Most notably they show that CnoX binds at a different site from GroES yet affects the kinetics of transition to the previously reported GroEL-GroES conformation. The cryoEM seems quite thorough and complete. The kinetics analysis is rudimentary but adequate to stimulate further work.
The gradual constriction of GroEL by GroES binding from state III to state IV is most intriguing in that it suggests a barrier that is created by CnoX and overcome by GroES. Although the GroES mobile loop is said to be more ordered in state IV, it is nevertheless presented as structured (with high b-factors) in state III. Can the authors compare the structures and point to differences that stabilize IV over III?

Kim et al, Response to Reviewer Comments (LSA-2026-03764-TR)

We would like to thank the reviewers for their time and constructive feedback on our manuscript.

Response to Reviewer #1 :

This paper describes the structures of four states of CnoX in complex with GroEL bound to an ATP analog and with or without GroES. One state has been described before but the others are new and informative. The revised version is much improved after various speculations and ATPase data have been removed. My only very minor comment is that most of the cited reviews in the paper are old (2001, 2011, 2016) and much has changed. I suggest adding/replacing with newer reviews.

We thank the reviewer for this helpful suggestion. We agree that several of the review articles cited in the original manuscript were outdated. Accordingly, we have revised the reference list by removing the older review articles and replacing them with more recent reviews that better reflect current advances in the field. We believe these updated references provide a more accurate and up-to-date context for our study.

Response to Reviewer #2 :

Kim et al. report new conformational states of GroEL formed in complex with redox enzyme CnoX and GroES. Most notably they show that CnoX binds at a different site from GroES yet affects the kinetics of transition to the previously reported GroEL-GroES conformation. The cryoEM seems quite thorough and complete. The kinetics analysis is rudimentary but adequate to stimulate further work.
The gradual constriction of GroEL by GroES binding from state III to state IV is most intriguing in that it suggests a barrier that is created by CnoX and overcome by GroES. Although the GroES mobile loop is said to be more ordered in state IV, it is nevertheless presented as structured (with high b-factors) in state III. Can the authors compare the structures and point to differences that stabilize IV over III?

We thank the reviewer for this insightful comment. To address this point, we compared the State III and State IV structures to identify structural differences that could account for the enhanced stability of State IV. Specifically, we analyzed the GroEL apical-domain interfaces and quantified the GroEL–GroES interface using PDBePISA. This analysis showed that, compared with State III, each corresponding GroEL–GroES subunit pair in State IV buries an additional ~157 Å^2 of surface area, resulting in a more extensive GroEL–GroES interface. Furthermore, this expanded interface is accompanied by a more compact arrangement of the GroEL apical domains, which strengthens the I305-centered hydrophobic network and seals the groove that accommodates CnoX in State III. Together, these structural differences provide a structural basis for the enhanced stability of State IV relative to State III. Accordingly, we have revised the corresponding section of the manuscript to describe these structural differences.

July 24, 2026

RE: Life Science Alliance Manuscript #LSA-2026-03764-TRR

Prof. Soung-Hun Roh
Seoul National University
School of Biological Science
1 Gwanakro
105-102
Seoul 08826
Korea, Republic of (South Korea)

Dear Dr. Roh,

Thank you for submitting your revised Research Article entitled "Structural interplay of the redox co-chaperone CnoX to GroEL/ES chaperonin". It is a pleasure to let you know that your manuscript is now accepted for publication in Life Science Alliance. Congratulations on this interesting work.

The final published version of your manuscript will be deposited by us to PubMed Central upon publication.

Your manuscript will now progress through copyediting and proofing. It is journal policy that authors provide original data upon request.

Your article will publish open access upon publication under a CC-BY license.

Reviews, decision letters, and point-by-point responses associated with peer-review at Life Science Alliance will be published, alongside the manuscript. If you do want to opt out of having the reviewer reports and your point-by-point responses displayed, please let us know immediately.

IMPORTANT: If you will be unreachable at any time, please provide us with the email address of an alternate author. Failure to respond to routine queries may lead to unavoidable delays in publication.

Scheduling details will be available from our production department. You will receive proofs shortly before the publication date. Only essential corrections can be made at the proof stage so if there are any minor final changes you wish to make to the manuscript, please let the journal office know now.

DISTRIBUTION OF MATERIALS:
Authors are required to distribute freely any materials used in experiments published in Life Science Alliance. Authors are encouraged to deposit materials used in their studies to the appropriate repositories for distribution to researchers.

You can contact the journal office with any questions, contact@life-science-alliance.org

Again, congratulations on a very nice paper. I hope you found the review process to be constructive and are pleased with how the manuscript was handled editorially. We look forward to future exciting submissions from your lab.

Sincerely,

Tim Fessenden
Scientific Editor
Life Science Alliance
