# Supplementary material for: Structural interplay of the redox co-chaperone CnoX to GroEL/ES chaperonin
Source: Life Sci Alliance. 2026 Aug 7;9(10):e202603764. doi: 10.26508/lsa.202603764 (PMC13451151; doi:10.26508/lsa.202603764)
Supplement: Supplementary file 2 [file LSA-2026-03764_TableS1.docx]

**Table S1. Cryo-EM data collection, processing, and refinement statistics**

|  | #1 State I  no symmetry  (EMDB-65953) | #2 State I with symmetry  (EMD-65877)  (PDB 9WCW) | #3 State II  (EMDB-65990)  (PDB 9WID) | #4 State III  (EMDB-65992)  (PDB 9WIF) | #5 State IV  (EMDB-65991)  (PDB 9WIE) |
| --- | --- | --- | --- | --- | --- |
| **Data collection and processing** |  |  |  |  |  |
| Magnification | 92000x | 92000x | 92000x | 92000x | 92000x |
| Voltage (kV) | 200 | 200 | 200 | 200 | 200 |
| Electron exposure (e–/Å^2^) | 40 | 40 | 40 | 40 | 40 |
| Defocus range (μm) | -0.8 ~ -1.6 | -0.8 ~ -1.6 | -0.8 ~ -1.6 | -0.8 ~ -1.6 | -0.8 ~ -1.6 |
| Pixel size (Å) | 1.08 | 1.08 | 1.08 | 1.1 | 1.1 |
| Symmetry imposed | C1 | C7 | C1 | C7 | C7 |
| Initial particle images (no.) | 1,292,437 | 1,292,437 | 1,292,437 | 1,869,887 | 1,869,887 |
| Final particle images (no.) | 214,141 | 214,141 | 29,790 | 143,513 | 133,737 |
| Map resolution (Å)      FSC threshold | 3.11  0.143 | 2.73  0.143 | 3.46  0.143 | 2.83  0.143 | 2.79  0.143 |
|  | | | | | |
| **Refinement** |  |  |  |  |  |
| Initial model used (PDB code) |  | 7YWY | 8BA8,  3QOU | 1AON,  3QOU | 1AON,  3QOU |
| Model resolution (Å)      FSC threshold |  | 2.73  0.143 | 3.46  0.143 | 2.83  0.143 | 2.79  0.143 |
| Model resolution range (Å) |  | 2.73 ~ 5.89 | 3.46 ~ 45.4 | 2.83 ~ 6.85 | 2.79 ~ 6.32 |
| Map sharpening *B* factor (Å^2^) |  | -101.9 | -46.4 | -91.9 | -96.4 |
| Model composition      Non-hydrogen atoms      Protein residues      Ligands |  | 64092  8568  42 | 64502  8610  35 | 69580  9289  35 | 64638  8666  35 |
| *B* factors (Å^2^)      Protein      Ligand |  | 72.89  69.68 | 146.35  90.57 | 117.70  70.40 | 98.18  89.75 |
| R.m.s. deviations      Bond lengths (Å)      Bond angles (°) |  | 0.630  0.004 | 1.210  0.006 | 1.300  0.006 | 1.225  0.006 |
| Validation      MolProbity score      Clashscore      Poor rotamers (%) |  | 1.15  3.62  0.02 | 2.11  21.82  0.01 | 2.17  20.76  0.48 | 2.02  16.43  0.01 |
| Ramachandran plot      Favored (%)      Allowed (%)      Disallowed (%) |  | 99.39  0.56  0.05 | 95.97  4.01  0.02 | 94.82  5.08  0.11 | 95.71  4.26  0.02 |
